# Autophagy—Unlocking New Dimensions in the Pathology and Treatment of Depression

**DOI:** 10.3390/cells14110795

**Published:** 2025-05-28

**Authors:** Qiang Luo, Yulong Zhao, Peng Ren, Xu Liu, Yingjian Chen, Qianru Ying, Junjie Zhou

**Affiliations:** 1 School of Rehabilitation Medicine, Gannan Medical University, Ganzhou 341000, China; luoqiang@gmu.edu.cn (Q.L.); zhaoyulong@gmu.edu.cn (Y.Z.); renpeng1@gmu.edu.cn (P.R.); liuxu@gmu.edu.cn (X.L.); chenyingjian1@gmu.edu.cn (Y.C.); yingqianru@gmu.edu.cn (Q.Y.); 2 Key Laboratory of Prevention and Treatment of Cardiovascular and Cerebrovascular Diseases of Ministry of Education, Gannan Medical University, Ganzhou 341000, China; 3 Ganzhou Key Laboratory of Rehabilitation Medicine, Ganzhou 341000, China

**Keywords:** depression, autophagy, pathophysiology, antidepressant

## Abstract

Depression is a widespread mental disorder whose impact on an individual’s health extends far beyond the psychological dimension. As a disease with a significant burden, the effective treatment of depression has become a major challenge for global public health. Although several hypotheses have been proposed for the pathogenesis of depression, its pathophysiological mechanisms remain complex and not yet fully understood. Recent studies suggest that dysfunctional autophagy may play an important role in the development of depression. Autophagy, as an important intracellular degradation mechanism, maintains neuronal function and health by removing excess proteins and damaged organelles. Current evidence suggests that the regulation of autophagic processes may provide new potential targets for the treatment of depression. In this paper, we review the pharmacological mechanisms of autophagy by different antidepressant drugs and the abnormal changes in autophagy in patients with depression and in multiple models. Importantly, we focus on the role of autophagy in different pathological mechanisms of depression and discuss current limitations as well as potential directions for future research.

## 1. Introduction

Depression is a serious mental illness characterized by persistent low mood, an absence of pleasure, and a wide range of cognitive and somatic symptoms. Globally, depression has become a leading cause of disability, with the number of people with depression worldwide increasing by nearly 50 per cent over the past 30 years, currently affecting approximately 264 million people [1]. The global burden of mental disorders was further exacerbated during the COVID-19 pandemic, with the number of people suffering from depression surging by 53 million, an increase of 27.6% [2]. Depression is not limited to mental health; it significantly increases the risk of cardiovascular disease, stroke, diabetes, and obesity, and has become a major public health challenge worldwide [3]. Currently, depression is classified into various subtypes based on symptom characteristics such as postpartum depression [4], seasonal depression [5], and psychotic depression [6]. Although researchers have proposed many hypotheses to explain the mechanism of depression, including the classical monoamine hypothesis, hypothalamic–pituitary–adrenal (HPA) axis hypothesis, neuroplasticity hypothesis, immunoinflammatory hypothesis, and neurogenesis hypothesis, the specific pathophysiological mechanisms of depression are still not fully understood [7,8,9]. And the treatments for different depression subtypes are based on different hypothesized mechanisms and therefore differ in their treatment approaches. For example, psychotic depression is usually treated with a combination of antidepressants and antipsychotics because it is accompanied by psychotic symptoms in addition to the exhibition of depressive symptoms [10]. In contrast, in seasonal depression, bright light therapy acts on depression by modulating circadian rhythms and affecting melatonin and serotonin secretion [11]. Currently, most antidepressant drug development is still based on the monoamine hypothesis, such as selective serotonin reuptake inhibitors (SSRIs), tricyclic antidepressants, serotonin and norepinephrine reuptake inhibitors, and monoamine oxidase inhibitors [12]. However, despite the fact that these medications are effective to some extent, about one-third to one-half of patients with depression still do not respond to multiple antidepressant medications, and among those in whom the medications are effective, common side effects such as headache, gastrointestinal distress, sexual dysfunction, and anxiety seriously affect patients’ quality of life [13]. In addition, the difference in the efficacy of bright light therapy in seasonal and non-seasonal depression still needs to be further verified [11]. Currently, the diagnosis of depression still lacks objective biomarker support [14]. Although genome-wide association studies have identified more than 200 genes associated with depression, these findings have not yet been translated into practically usable clinical diagnostic tools [15]. Therefore, there is an urgent need to explore new research directions in order to provide guidance for clinical diagnosis and to improve the effectiveness of treatment.

Autophagy is the process by which cells use lysosomes to degrade their own proteins and damaged organelles, and it is essential for maintaining cellular homeostasis and responding to nutrient deficiencies and stress. Since neurons are highly specialized cells and do not possess the ability to continue cell division, they are particularly dependent on the autophagy pathway to remove excess proteins and damaged organelles [16]. Thus, autophagy plays a key role in both the developmental stages of the brain and in adulthood, participating in the development and refinement of neuronal axons, dendrites, and synapses. In recent years, more and more clinical and preclinical studies have shown that abnormal autophagy is closely related to the occurrence and development of depression and that the regulation of autophagy plays an important role in depression [17]. Therefore, in-depth exploration of the mechanisms of autophagy pathways in depression is important for the development of novel therapeutic strategies.

In this review, we provide a comprehensive overview of general knowledge of autophagy mechanisms and describe the relationship between depression and aberrant autophagy in individuals as well as in animal and cellular models. We also focus on the role of autophagy in different pathomechanisms of depression and summarize the pharmacological mechanisms of different drugs based on autophagy modulation to improve depression, providing a scientific basis for autophagy-targeting antidepressants in the clinic in the future.

## 2. Overview of Autophagy

**Figure 1:**
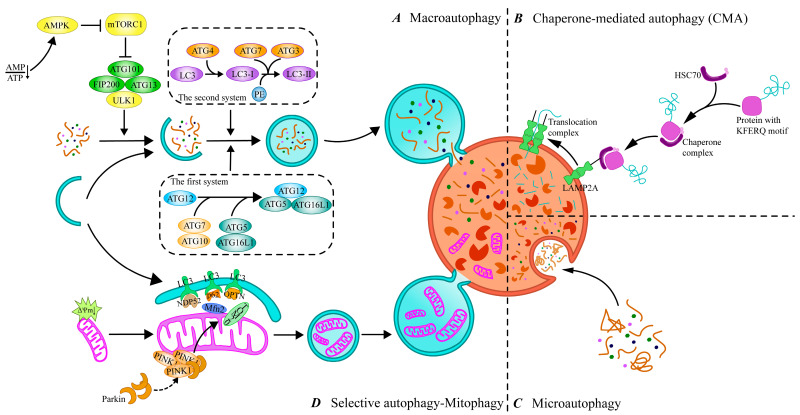
Different types of autophagy and processes. (**A**) Macroautophagy. The process of macroautophagy includes initiation, autophagosome formation, fusion, and degradation, which is the main type of autophagy. (**B**) Chaperone-mediated autophagy (CMA). CMA recognizes KFERQ-like motif proteins that interact with cytosolic Hsc70 and its chaperones to make them target lysosomes. (**C**) Microautophagy. Microautophagy delivers targets to lysosomes through invagination of lysosomal membranes. (**D**) Selective autophagy—mitophagy. Mitophagy targets the removal of damaged mitochondria through the autophagy pathway. ATG, autophagy-related genes; AMP, adenosine monophosphate; ATP, adenosine triphosphate; AMPK, AMP-activated protein kinase; mTORC1, mTOR complex 1; ULK1, unc51-like autophagy-activating kinase; FIP200, FAK-family interacting protein of 200 KD; HSC70, Heat Shock 70 protein; OPTN, Optineurin; PE, phosphatidylethanolamine; LAMP2A, lysosomal-associated membrane protein 2A; LC3, microtubule-associated protein 1 light chain 3; NDP52, Nuclear Dot Protein 52; Mfn2, mitofusin 2; PINK1, PTEN-induced putative kinase 1; TOM20, Translocase of outer mitochondrial membrane 20.

Currently, autophagy can be classified into three main types according to how intracellular substances are transported to lysosomes: macroautophagy, chaperone-mediated autophagy (CMA), and microautophagy (Figure 1). Macroautophagy is the classical form of autophagy, in which cytoplasmic material is encapsulated by autophagosomes in a double-membrane structure and transported to the lysosome for degradation by fusion with the lysosome. CMA is a form of selective autophagy, in which cargo proteins containing specific pentapeptide motifs are recognized by a molecular chaperone, Heat Shock Cognate 70 (Hsc70), and guided to the lysosome. Microautophagy, on the other hand, directly wraps the contents of the cytoplasm for degradation through the invagination of the lysosomal membrane. With further research on autophagy, it has been found that autophagy can degrade some special organelles or macromolecules through selective degradation, which is called selective autophagy, with forms such as mitophagy, ribophagy, pexophagy, and so on [18]. Among these, mitophagy, as the most widely studied form of selective autophagy, has been demonstrated to play an important role in maintaining intracellular homeostasis and preventing cellular damage and the development of related diseases. In this paper, we will focus on the mechanisms of macroautophagy, CMA, microautophagy, and mitophagy to discuss their roles in intracellular material degradation and homeostasis maintenance.

### 2.1. Macroautophagy

Macroautophagy is the most studied form of the autophagy pathway, hereafter referred to as “autophagy”. The process of autophagy is finely regulated at multiple levels to ensure a balance between the synthesis and degradation of intracellular substances and the use and recycling of resources. This regulatory network involves multiple signaling pathways to ensure cellular homeostasis under different physiological or pathological conditions. It has been shown that more than 40 autophagy-associated genes (ATGs) have been identified in yeast, and the roles of the proteins encoded by these genes in the autophagy process have been intensively explored, with most of these proteins also having corresponding homologs found in mammalian cells [19]. The initiation of autophagy is tightly linked to the mammalian target of rapamycin complex 1 (mTORC 1) and unc 51-like autophagy-activated kinase 1 (ULK 1) complexes [20]. Upon cellular stress, the intracellular activation of the ULK1 complex through the inhibition of mTORC1 subsequently initiates PI3K complex function, that is, the generation of phosphatidylinositol 3-phosphate (PI3P) via vesicular protein sorting 34 (VPS34), which is involved in the nucleation of phagocytic vesicles [21]. In addition, the accumulation of PI3P at the phagocytic vesicle assembly site leads to the recruitment of autophagy-related genes and promotes the extension and closure of autophagosomes [22]. Among them, ATG12 binds to ATG5 via the E1 ubiquitin-activating enzyme ATG7 and the E2 ubiquitin transferase ATG10, and then forms a large complex with the ATG16L1 composition via non-covalent interactions [19]. In addition, ATG4 shears the carboxyl terminus of LC3, one of the mammalian ATG8 homologs, to produce LC3-I, which is converted to LC3-II bound to the autophagosome membrane to form mature autophagosomes via ATG7 and ATG3 binding to phosphatidylethanolamine (PE) [23]. Then, mature autophagosomes fuse with lysosomes to form autophagic lysosomes to complete the degradation and recycling of contents.

### 2.2. Chaperone-Mediated Autophagy (CMA)

CMA achieves its purpose by recognizing the KFERQ-like targeting motif on the substrate protein, which carries the motif and binds to HSC70 to form a complex. HSC70 not only recognizes and binds the substrate protein but also powers the substrate protein through its ATPase activity, which directs the complex to the lysosomal membrane to bind to lysosome-associated membrane protein 2A (LAMP2A) [24]. LAMP2 is an important membrane protein on lysosomes, and three protein isoforms, LAMP2A, LAMP2B, and LAMP2C, exist, of which only LAMP2A is involved in CMA, and it is an essential molecule for the fusion of autophagosome and lysosome [25]. Upon the binding of HSC70 to LAMP2A, the LAMP2A monomer assembles into a multimeric structure by recruiting multiple LAMP2A molecules to form a transport complex that provides substrate proteins with access to the lysosome [26]. It was found that high-fat diet (HFD)-induced obesity may lead to depression-like behavior in mice by inhibiting autophagy [27]. Further studies indicated that HFD alters lysosomal membrane lipid composition, decreases LAMP2A stability, and inhibits CMA [28]. It can be seen that the expression of LAMP2A is closely related to the activity of CMA, and its polymerization state and interaction with HSC70 directly affect the transport efficiency of substrate proteins, and alterations in its function may affect the overall efficiency of CMA and the homeostasis of intracellular proteins. In addition, the regulatory process of CMA is closely related to multiple signaling pathways. For example, in the neurodegenerative disease Parkinson’s disease, the p38 MAPK pathway exacerbates disease progression by promoting microglia activation through inhibiting the degradation of NLRP3 inflammatory vesicles by CMA [29]. This finding suggests that CMA is not only involved in intracellular protein quality control but may also play an important role in neuroinflammatory responses.

### 2.3. Microautophagy

Compared with macroautophagy and CMA, microautophagy has been relatively understudied. Microautophagy mainly forms vesicles through localized depressions and invaginations in the cell membrane, thereby encapsulating and endocytosing extracellular fluid and solutes into the cell [30]. Unlike macroautophagy and CMA, the process of microautophagy mainly relies on the direct remodeling of the lysosomal membrane. In this process, specific membrane proteins present on the lysosomal membrane play a key role, which are not only involved in morphological changes in the membrane but also responsible for recognizing and targeting cytoplasmic components that need to be degraded [31]. For example, during microautophagy, certain proteins can be labeled by a K63-type ubiquitination modification that is recognized by the endosomal sorting complex required for transport (ESCRT), which in turn facilitates the targeted transport of these proteins to the lysosome for degradation [32]. Microautophagy can not only target degradation through the ubiquitination mechanism but also selectively phagocytose specific intracellular lipid signals by recognizing the component, that is, microliphagy [33]. Additionally, selective microautophagy can also be performed on a variety of intracellular components such as mitochondria, the endoplasmic reticulum, and the nucleus [34]. In micromitochondrial autophagy, damaged mitochondria release mitochondria-derived vesicles (MDVs), which are rich in oxidized mitochondrial proteins, and MDVs ultimately become multivesicular vesicles that fuse with lysosomes to complete degradation [35]. Although some progress has been made in this field in recent years, gradually revealing the important role of microautophagy in intracellular material degradation and homeostasis maintenance, the exploration of microautophagy is still very different compared to that of macroautophagy and CMA.

### 2.4. Mitophagy

Mitophagy, as a central mechanism for the selective removal of damaged mitochondria, is essential for mitochondrial quality and quantity control [36]. Mitophagy is usually classified into two main types: PINK1/Parkin-dependent and non-PINK1/Parkin-dependent. In PINK1/Parkin-mediated mitophagy, PTEN-inducible kinase 1 (PINK1) is blocked from entering the inner mitochondrial membrane when the mitochondrial membrane potential decreases and steadily accumulates in the outer mitochondrial membrane (OMM). PINK1 activates and recruits Parkin, an E3 ubiquitin ligase, to the outer mitochondrial membrane through autophosphorylation [37]. Activated Parkin then forms ubiquitin chains by ubiquitinating mitochondrial outer membrane proteins (e.g., TOM20, Mfn2), thereby labeling damaged mitochondria and facilitating the recruitment of autophagy junction proteins such as p62/SQSTM1, OPTN, and NDP52 [38,39]. These receptor proteins bind to LC3 via their LC3-interacting region (LIR), which initiates the autophagosomal encapsulation and degradation of damaged mitochondria [40]. In addition to the PINK1/Parkin-dependent pathway, recent studies have identified multiple non-PINK1/Parkin-dependent pathways in which specific receptor proteins also recognize and target damaged mitochondria for autophagy. Examples include NIX (Nip3-like protein X), BNIP3 (Bcl2-interacting protein 3), and FUNDC1 (FUN14 structural domain-containing 1) [41]. These receptor proteins directly interact with LC3 or γ-aminobutyric acid receptor-associated protein (GABARAP) through their LIR motifs to promote autophagic clearance in mitochondria [42]. Among them, the expression levels of BNIP3 and NIX are tightly regulated under normal cellular conditions, but in a hypoxic environment, hypoxia-inducible factor 1α (HIF-1α) is able to transcriptionally up-regulate their expression, which promotes mitochondrial removal and helps the cells to adapt to the low-oxygen environment [43]. In addition, FUNDC1 tightly links mitochondrial quality control to the autophagy process by regulating the process of mitochondrial fission and fusion, ensuring that cells can effectively remove damaged mitochondria [44].

Autophagy achieves intracellular homeostasis through a multilevel regulatory network, and the diversity of its types and mechanisms reflects the flexible strategies of cells to cope with stress. A large number of studies have found that the above four types of autophagy are involved in the pathological process of depression. However, despite great progress in understanding the mechanisms of autophagy, many questions remain.

## 3. Molecular Mechanisms of Autophagy and Its Abnormalities in Depression

### 3.1. Autophagy Dysfunction in Depression

#### 3.1.1. Autophagy Dysfunction in Patients with Depression

Autophagy dysfunction has become an important direction of current interest in the study of depression, especially in terms of changes in autophagy-related proteins and their regulatory pathways. An analysis of postmortem prefrontal cortex (PFC) tissues from patients with depression by single-cell sequencing revealed a significant decrease in the proportion of astrocytes and a close relationship between the inhibition of their autophagic pathway and the development of depression [45]. In addition, postmortem human brain microarray analysis showed a significant increase in the transcript levels of several autophagy-related genes such as ATG5, ATG6, ATG7, and ATG12 [46]. Meanwhile, another study showed that the mRNA and protein levels of p62 were significantly increased in the blood of patients with depression, and this change was further verified in an animal model [47]. In addition, a study examined the levels of NIX and LC3 in the peripheral blood of patients with depression by qPCR and found that the levels of these two autophagy-related molecules were significantly reduced, further suggesting that dysfunction in autophagy may be a common feature of depression [48]. These findings suggest that the abnormal changes in the expression of autophagy-related molecules in patients with depression are closely related to dysfunction in autophagy, which may provide new perspectives on biomarkers of depression. Although a large number of studies have explored these changes in autophagic pathways and molecular markers, there is still some inconsistency between the results of different studies. This discrepancy may be related to differences in patients, with them being at different stages of the disease, and the clinical manifestations and biomarkers of depression may change as the disease progresses. Therefore, future studies need to further explore, in depth, the role of autophagy and its mechanisms in different disease stages of depression, with a view to providing a new theoretical basis for the early diagnosis and treatment of depression.

#### 3.1.2. Autophagy Dysfunction in Models of Depression

In the study of pathological mechanisms of depression, autophagy dysfunction is believed to play a key role in the onset and development of the disease. A large number of studies have shown that abnormalities in the autophagic process have a profound impact on the pathogenic mechanisms of depression. For example, the expression of autophagy-related proteins LC3-II and Beclin-1 was significantly reduced in brain tissue in a mouse model of LPS-induced depression [49]. However, another study showed that corticosterone (CORT) significantly activated cellular autophagy by damaging hippocampal neurons in newborn neurons, which was accompanied by elevated levels of LC3-II and ATG5 proteins [8]. Notably, the expression of ULK1, a key regulator in the autophagy process, did not show significant changes during this process, suggesting that the activation of the autophagy pathway may have different mechanisms in different depression models [8]. Socio-environmental factors, especially chronic stressful stimuli, are important external factors in the development and progression of depression. In order to study the effects of stress on depression, scientists have developed a variety of stress-induced animal models of depression, such as CUMS, the chronic social defeat stress (CSDS) model, and the learned helplessness (LH) model [50]. It was shown that in the CUMS-induced mouse model, there was a significant decrease in LC3 and Beclin-1 expression in brain tissue, along with increased levels of p62 and mTOR expression [51]. These changes suggest that both the inhibition of autophagy and the activation of the mTOR signaling pathway may be involved in the stress-induced depression model. In addition, impaired mitophagy was observed in this model, as evidenced by a decrease in the number of autophagosomes in the hippocampal region and a down-regulation in the expression of autophagy-related proteins such as LC3-II/I, ATG5, PINK1, and Parkin [52]. Similar results were also seen in the CSDS-induced mouse model of depression, where the expression of autophagy-related proteins such as LC3-II/I, Beclin-1, ATG5, and ATG7 was significantly decreased in the hippocampus after 10 days of stress exposure [53]. In the pathogenesis of depression, translocator protein (TSPO), as an important regulator of mitophagy, has also received extensive attention. TSPO inhibits PINK1/Parkin-mediated mitophagy via a voltage-dependent anion channel (VDAC1), thereby limiting the ubiquitination of related proteins [54]. In the LH mouse model, it was found that the expression of mitophagy-associated proteins such as TSPO, VDAC1, PINK1, and Beclin-1 was significantly decreased [55]. In addition, the expression levels of LC3-II, PINK1, and Parkin were also significantly decreased in LPS- and ATP-treated BV2 cells, whereas the expression of proteins such as p62, TOM, and TIM was increased. Further immunofluorescence colocalization analysis showed that the colocalization of the mitochondrial fluorescent probes MitoTracker and LC3-II was reduced, suggesting that mitophagy function was inhibited [56]. Autophagic flux and mitophagy were similarly inhibited in LPS-induced rat primary astrocytes, as evidenced by decreased LC3 levels and increased p62 levels [57]. In CORT-treated HT22 cells, a reduction in the number of autophagosomes was also observed, as well as a significant decrease in the mRNA expression levels of PINK1, Parkin, ATG5, and LC3 [52]. The close association of autophagy with the pathogenesis of depression is further supported.

Overall, these findings suggest that cellular autophagy is commonly impaired in a variety of depression models. The impairment of cellular autophagy, an important mechanism of cellular stress response, may lead to the impairment of normal cellular function, which may have serious implications for overall function. These findings provide new perspectives for further understanding the pathogenesis of depression and highlight the potential role of autophagy function in the development of depression. (Detailed information on autophagy-related changes in different depression models is shown in Table 1).

### 3.2. Autophagy Regulatory Pathways

#### 3.2.1. mTOR-Dependent Pathways

The mammalian target of rapamycin complex (mTORC1/mTORC2)-dependent pathway plays a crucial role in the regulation of cellular metabolism, autophagy, and neurological function and has been the focus of several studies [58]. Mammalian target of rapamycin (mTOR) is a serine/threonine kinase that plays a central role in a variety of biological processes as a key regulator of intracellular metabolism and autophagy. Specifically, mTORC1 acts at different stages of autophagy by phosphorylating several important molecules in the process [59,60]. It has been found that the inhibition of mTORC1 activates the AMPK pathway and thus promotes mitochondrial function, which is essential for neuronal energy metabolism [61]. In addition, inactivation of mTORC1 is able to impede mitophagy while hindering mitochondrial function in neurons, thus worsening neuronal survival; this mechanism has potential applications in the treatment of neurodegenerative diseases [62]. In the nervous system, mTORC1 is not only crucial for neuronal energy metabolism but also plays a central role in synaptic plasticity and the process of learning and memory. Depression is closely associated with altered synaptic plasticity, and long-term chronic stress causes stress hormone disruption, which in turn affects synaptic plasticity, decreasing the number of synapses in several regions of the brain and leading to hippocampal neuronal atrophy [63]. The role of mTORC1 is particularly prominent in synaptic plasticity, where synaptic plasticity and dendritic spine formation depend on the synthesis of new proteins from scratch. However, mTORC1 can play a crucial role in the biological processes of learning and long-term memory by regulating protein synthesis through phosphorylation mediating the processes involved [64]. Recent studies further reveal that genetic deletion of mTORC1 alleviates the increase in excitatory synaptic transmission due to the loss of the Pten gene and reduces the overgrowth of neuronal cytosolic dendrites and spines [65]. In addition, in the Rptor-deficient mouse model, the deletion of the mTORC1 complex resulted in defects in axonal innervation compartments of dopamine neurons in the midbrain, further suggesting the importance of mTORC1 in neural development [66]. Recently, the potential of the mTORC1 pathway in the treatment of psychiatric disorders has also attracted much attention. For example, fluoxetine promotes autophagy and reverses depressive-like behaviors by targeting mTOR and modulating p-mTOR/mTOR levels in a rat model of olfactory bulbectomy [67]. These studies not only open up new horizons for the application of mTORC1 in neuropsychiatric disorders but also provide a theoretical basis for future drug development.

#### 3.2.2. mTOR-Independent Pathways

In the non-mTOR-dependent autophagy pathway, AMPK and extracellular signal-regulated kinase (ERK), among others, play important roles in the regulation of autophagy; AMPK, as a key energy sensor, is able to respond to the energy status of the cell by sensing the changes in the intracellular AMP/ATP ratio and thus activate autophagy in response to cellular stress [68]. Studies have shown that the activity of the AMPK-ULK1-FUNDC1 signaling pathway is significantly inhibited in the neurotrophic tyrosine kinase receptor 1 (NTRK1) knockout mouse model and that this inhibition blocks mitophagy and leads to dysfunctional mitochondria, enhanced oxidative stress, and synaptic damage in hippocampal neurons, ultimately leading to cognitive deficits [69]. However, it is worth noting that contrary to the conventional view that AMPK promotes autophagy, the activation of AMPK in the presence of amino acid deprivation inhibits autophagy [70]. It was further shown that AMPK could inhibit the initiation of autophagy by phosphorylating the Ser556 site of ULK1 [71]. This finding reveals that the mechanism by which AMPK acts on autophagy is more inclined to inhibit than activate autophagy in an amino acid-deficient environment. ERK1/2, as an important member of the mitogen-activated protein kinase (MAPK) family, plays a key role in a variety of pathological processes, especially in the development of diseases such as depression [72]. It has been found that the microbial metabolite urolithin A can directly bind to ERK1/2 and promote its activation, followed by the phosphorylation and activation of the autophagy initiation factor ULK1, thereby initiating the autophagy process [73]. In addition, it has been shown that mitogen-activated protein kinase phosphatase-1 (MKP-1) activates autophagy in the hippocampal region by inhibiting the phosphorylation of ERK1/2, which in turn promotes the accumulation of LC3-II and the impairment of synaptic plasticity, a process that exacerbates the manifestation of depressive-like behaviors in the chronic unpredictable stress (CUMS) model of rats [74]. In addition to AMPK and ERK, Ca^2+^ signaling, reactive oxygen species (ROS), and JNK–Beclin-1 signaling pathways also play important roles in the regulation of the mTOR-independent autophagy pathway [75]. These signaling pathways synergistically regulate the onset and progression of autophagy by interacting with different molecules related to autophagy, further revealing the complexity and diversity of autophagy regulation.

### 3.3. Autophagy Markers and Depression

#### 3.3.1. p62

As a multifunctional protein, p62 acts as an autophagy receptor that recognizes and binds ubiquitinated proteins and delivers them to the autophagosome for degradation [76]. Specifically, p62 initiates autophagosome nucleation and elongation through the vesicle aggregation of ubiquitinated substrates and the recruitment of autophagy-related proteins and membrane sources [77]. It has been shown that the S-lipoylation modification of p62 significantly enhances its binding affinity to autophagosome membranes by increasing its hydrophobicity, thereby regulating the recruitment of p62 vesicles to autophagosomes, forming a dynamic regulatory loop [78]. This process makes the role of p62 in autophagy more fine-grained and regulatable. In addition, it has been found that lipopolysaccharide (LPS) or dextran sodium sulfate (DSS) exacerbates depression- and anxiety-like behaviors induced by prolonged restraint stress in mice and is closely associated with decreased p62 expression levels [79]. Further studies have shown that p62 overexpression can alleviate depression- and anxiety-related behaviors by improving mitochondrial function in the hippocampus, as evidenced by a reduction in anxiety and a significant increase in exploratory activity in mice tested in both the open-field and elevated plus maze tests [80]. In contrast, p62 deficiency resulted in anxiety, depression, and a loss of working memory, accompanied by decreased serum brain-derived neurotrophic factor (BDNF) levels [81]. These results suggest that p62 is involved in the onset and development of mood disorders through the regulation of autophagy and mitophagy and, in particular, plays a key role in the modulation of symptoms such as anxiety and depression. However, although studies have revealed the important role of p62 in autophagy, the specific effects of p62 deletion or overexpression on mitochondrial autophagic fluxes still need to be further investigated. In particular, the regulatory mechanisms of p62 may play different roles in different disease processes, and its potential efficacy in mood disorders is still worth exploring.

#### 3.3.2. Neighbor of BRCA1 Gene 1 (NBR1)

Similarly to p62, NBR1 is an autophagy receptor that was discovered shortly after p62. NBR1 in animals is an evolutionarily conserved selective autophagy receptor that plays a key role in the specific selection and recognition of autophagic substrates [82]. NBR1 has similar structural domains to p62, containing a ubiquitin-associated domain structural domain and two LIR motifs, as well as the Phox and Bem1 (PB1) structural domain and the ZZ zinc finger domain, in which the PB1 structural domain binds to p62 [83]. In addition, NBR1 is able to recruit another autophagy receptor protein, TAX1BP1, to aggregates and the postural protein FIP200, thereby promoting autophagosome formation [84]. Post-stroke depression (PSD) is the most common psychiatric complication after stroke, and elevated mRNA and protein expression of NBR1 in hippocampal tissues, along with elevated ULK1 expression, was found in a rat model of PSD constructs [85]. Furthermore, in the HIF-1α knockout rat model of PSD, depressive-like behavior was improved; however, NBR1 overexpression reversed this effect [86]. The peroxisome is an important organelle for maintaining intracellular redox homeostasis, and the level of ROS within it correlates with the level of oxidative stress in vivo [87]. It is well known that free radicals generated by oxidative stress can damage cellular structure and function, leading to neuronal damage or death, thereby increasing the risk of depression. NBR1 expression was found to increase peroxisome-targeted lysosomes for degradation through the autophagy pathway to control their quality and quantity [88]. In summary, NBR1 can improve oxidative stress levels and depression-like behavior in vivo through autophagy, and it is likely to be a potential target for the treatment of depression.

#### 3.3.3. Mitophagy-Specific Markers (e.g., PINK1/Parkin)

PINK1 is a serine/threonine kinase that is widely involved in mitochondrial quality control and plays a crucial role in regulating mitophagy in particular. In the healthy mitochondrial state, PINK1 is rapidly degraded by the mitochondrial protease PARL, thereby maintaining normal mitochondrial function [89]. However, the dysregulation of the PINK1/Parkin signaling pathway is closely associated with the development of a wide range of diseases, particularly neuropsychiatric disorders. For example, the PINK1/Parkin-mediated inhibition of mitochondria has been found in Alzheimer’s disease mice and β-amyloid-induced cellular models of SH-SY5Y cells, along with the exacerbation of neuroinflammation and cellular focal death [90]. In a rat model of depression–insomnia co-morbidity with CUMS combined with sleep deprivation, dysfunction in the PINK1/Parkin signaling pathway led to impaired mitophagy in the pineal gland, which in turn triggered a significant reduction in 5-HT levels and an increase in the release of markers of oxidative stress (ROS, Malondialdehyde). Meanwhile, the activation of NF-κB further promotes the release of the inflammatory factor IL-1β and ultimately triggers depressive behaviors [91]. In addition, defects in the PINK1/Parkin pathway can lead to the accumulation of damaged mitochondria, which further exacerbates the dysfunction of the electron transport chain and defects in ATP synthesis, thus triggering an energetic crisis in neurons and worsening depressive symptoms [92]. It has been shown that by overexpressing PINK1 or Parkin, the down-regulation of mitophagy-related proteins can be reversed, the Bax/Bcl-2 ratio can be improved, and mitochondrial damage can be reduced, which in turn improves behavioral abnormalities, such as immobility time in the forced swimming test and tail suspension test [91]. Notably, melatonin analogs contribute to the restoration of mitophagy and amelioration of behavioral abnormalities associated with cognitive deficits through the activation of the AMPK/PINK1 signaling pathway, which offers a new potential strategy for the treatment of depression [93]. Thus, the dysregulation of the PINK1/Parkin signaling pathway plays a key role in the onset and progression of depression, and the enhancement of PINK1/Parkin-mediated mitophagy may be a novel means of alleviating depression.

## 4. Interaction Between Autophagy and Pathological Mechanisms of Depression

**Figure 2:**
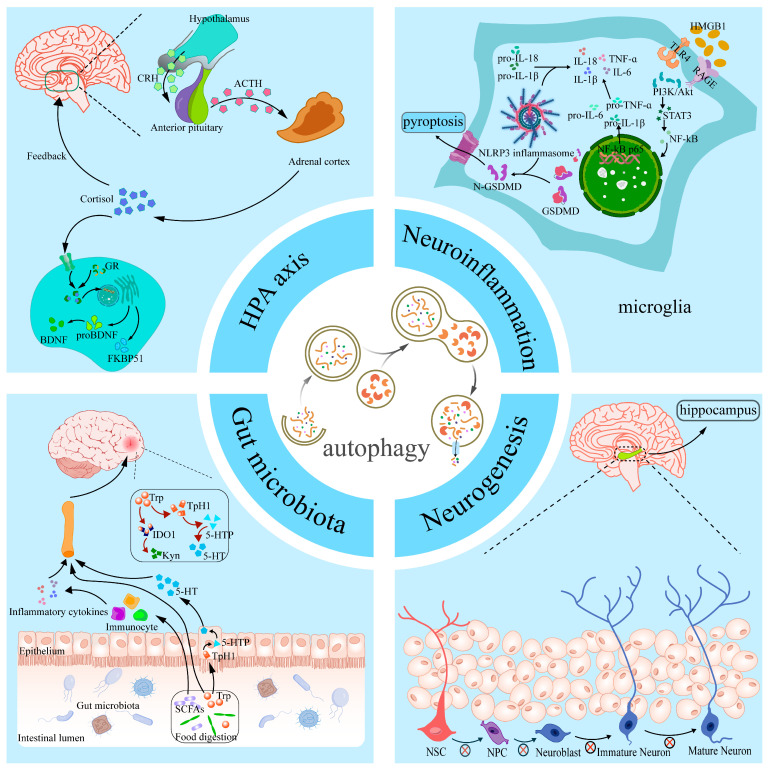
Overview of different pathological mechanisms linking autophagy and depression. Autophagy affects the development of depression by participating in neuroinflammation, hippocampal neurogenesis, the HPA axis, and gut microbiota. HMGB1, high-mobility group box-1 protein; TLR4, Toll-Like Receptor 4; RAGE, Receptor for Advanced Glycation End-Products; PI3K/AKT, phosphatidylinositol 3kinase/protein kinase B; STAT3, Signal Transducer And Activator Of Transcription 3; NF-κB, Nuclear factor-kappa B; IL-18, Interleukin-18; IL-6, Interleukin-6; IL-1β, Interleukin-1β; TNF-α, Tumor Necrosis Factor-α; GSDMD, Gasdermin D; NLRP3, NOD-like receptor protein 3; NSC, neural stem cell; NPC, neural progenitor cell; CRH, corticotropin-releasing hormone; ACTH, Adrenocorticotropic Hormone; GR, glucocorticoid receptor; BDNF, brain-derived neurotrophic factor; FKBP51, FK506-binding protein 51; Trp, tryptophan; TpH1, Tryptophan Hydroxylase 1; SCFAs, Short-chain fatty acids; Kyn, kynurenine; 5-HT, 5-Hydroxytryptophan; IDO1, indoleamine 2,3-dioxygenase 1.

More and more studies have shown that the autophagy signaling pathway plays a key role in the onset and development of depression. Animal model studies have shown that dysfunction in autophagy has been widely observed in commonly used depression induction models such as CSDS [53], LPS [94], and CORT [8]. Through these studies, it is suggested that the dysregulation of autophagy may be an important component of the pathology of depression. Through a comprehensive analysis of relevant datasets, researchers have identified four potential autophagy-associated genes that are considered diagnostic markers for depression [95]. Further clinical studies have shown that changes in the levels of the autophagy marker Beclin-1 are strongly associated with the response to medication for depression and that Beclin-1 may serve as an independent predictor of the efficacy of medication for depression [96]. These pieces of evidence fully indicate that the role of autophagy in depression cannot be ignored, and more basic and clinical studies are urgently needed to further elucidate its specific mechanisms. With the deepening of research on depression, more and more evidence suggests that autophagy is involved in multiple pathophysiological pathways of depression, including neuroinflammation, neurogenesis, dysbiosis of gut microbiota, and functional abnormalities of the HPA axis (Figure 2).

### 4.1. Autophagy and Neuroinflammation in Depression

Numerous studies have shown that neuroinflammation plays a key role in the development of depression [97]. Specifically, research data show that depression is often accompanied by elevated levels of pro-inflammatory cytokines in the blood and cerebrospinal fluid of patients with depression [98]. The activation of the NLRP3 inflammasome has been observed in several animal models of depression, such as LPS, stress, and ovariectomy induction models [99,100,101]. The NLRP3 inflammasome consists of the sensor molecule NLRP3, apoptosis-associated speck-like protein containing a caspase recruitment domain ASC, and the effector molecule Caspase-1, which regulates pro-inflammatory cytokine levels in response to cellular stimulation. Caspase-1 regulates the secretion of pro-inflammatory cytokines such as IL-1β and IL-18 [102]. The activation of NLRP3 and the release of IL-1β and IL-18 may be involved in the pathogenesis of depression through multiple mechanisms. For example, activated NLRP3 inflammatory vesicles can promote depression by prompting microglia to activate indoleamine 2,3-dioxygenase (IDO) and exacerbate neuroinflammation through kynurenine pathway signaling [103]. It has been shown that IL-1β and IL-18 released upon the activation of NLRP3 inflammatory vesicles may be key molecules in triggering the immune response in depression and further exacerbate the pathological process through Gasdermin D (GSDMD)-mediated cellular pyroptosis response [102]. Notably, the activity of NLRP3 inflammatory vesicles is regulated by autophagy. It has been shown that autophagy induction significantly inhibits the assembly of the NLRP3-ASC–Caspase-1 complex, thereby reducing the overproduction of pro-inflammatory cytokines [104]. For example, in a mouse model of DSS-induced ulcerative colitis, NLRP3 inflammatory vesicle levels were significantly elevated, accompanied by the emergence of depressive-like behavior [105]. Microglia are the most abundant immune cells in the CNS, and their activation is modulated by inflammatory signals released by a variety of neurons, which in turn drive enhanced neuroinflammatory responses [106]. In a mouse model of chronic mild stress exposure, immunofluorescence staining revealed the significant activation of Iba-1-positive microglia in the hippocampus and cortex, as well as significantly elevated expression levels of pro-inflammatory microglia markers such as IL-1β, TNF-α, and IL-6, suggesting phenotypic shifts in microglia [107]. In addition, preclinical studies have found that the HMGB1/STAT3/p65 signaling axis plays an important role in the activation of microglia and the regulation of their autophagy levels in the prefrontal cortex of depressed mice [108]. In summary, autophagy not only reduces the overproduction of pro-inflammatory cytokines by inhibiting the assembly of NLRP3 inflammatory vesicles but also plays a key role in the anti-inflammatory process by regulating the activity of immune cells.

### 4.2. Autophagy and Neurogenesis in Depression

Neurogenesis is the process by which neural stem cells (NSCs) or neural progenitor cells (NPCs) differentiate to produce neurons that migrate to and make synaptic connections in specific functional regions of the brain to form neural networks and perform neural functions. Neurogenesis in mammals occurs primarily in two specific regions: the subventricular zone and the subgranular zone of the dentate gyrus of the hippocampus. Neurons generated in these regions migrate to the olfactory bulb and are involved in the processing of olfactory information and the functional regulation of other brain regions [109]. In recent years, a growing body of research has shown that reduced neurogenesis is closely related to the pathogenesis of depression [110]. When neurogenesis in the hippocampus is impaired, the neuroendocrine regulation of stress in the hippocampus will be impaired, which leads to a lowering of the stress threshold, which in turn exacerbates the emergence of a depressive-like phenotype [111]. In addition to this phenomenon found in depression, in Parkinson’s disease, we have found that neurotrophins are specific to the promotion of neurogenesis in the brain and could have potential in the treatment of Parkinson’s disease as a complement to cellular replacement therapies [112]. The relationship between autophagic processes and neurogenesis has also been extensively studied in models of depression. In mice subjected to CORT to simulate psychological stress, autophagic death of NSCs was found to be severely compromised as well as the integrity of neurogenesis [113]. Nuclear Receptor-Binding Factor 2 (NRBF2), an important component of the PI3K complex, plays an important role in autophagy [114]. It was found that in chronic stress model mice, the level of NRBF2 in the hippocampal dentate gyrus was significantly decreased, leading to abnormal autophagy function in the NSCs in this region, which in turn resulted in the depletion of the NSCs and damage in neurogenesis [114]. In addition, BDNF plays an important role in neurogenesis and mood regulation. BDNF not only promotes neuronal survival by regulating apoptosis but also plays a role as an antidepressant by promoting neurogenesis. In BDNF-administered animal models, researchers have found that BDNF has a significant antidepressant effect [115]. In a CORT-induced depression model, it was found that increasing the expression of ATG5 could lead to overactive neuronal autophagy, which would lead to the significant degradation of BDNF, thus hindering the conversion of NSCs to mature neurons. Conversely, reducing ATG5 expression in neurons was able to alleviate this pathology and improve depression-like behavior in mice [8].

### 4.3. Autophagy and Gut Microbiota in Depression

In recent years, a growing body of research has revealed a strong link between gut microbiota and depression. The gut microbiota is involved in the onset and development of depression by influencing the function of the central nervous system through the gut–brain axis (GBA) [116]. Studies have shown significant changes in the composition of the gut microbiota in patients with depression, commonly characterized by a reduction in the diversity of the gut microbiota and changes in the abundance of certain specific microbiota [117]. For example, some studies have found that the abundance of pro-inflammatory bacteria is significantly increased in patients with depression, particularly the relative abundance of taxa such as Ruminococcus, Eggerthella, Enterobacteriaceae, and Proteobacteria, and that these changes in the bacterial microbiota may act through a number of mechanisms, the most notable of which is by promoting intestinal and systemic inflammatory responses, thereby affecting brain function and mood regulation [118]. In addition, GBA interaction mechanisms involve multiple metabolic pathways. For example, one study found that the composition of the gut microbiota was significantly altered in a mouse model of CRS, and the study further observed that bacteria such as Enterorhabdus and Parabacteroides were negatively correlated with kynurenine (Kyn) levels in the brain [119]. The Kyn metabolic pathway is one of the major branches of tryptophan (Trp) metabolism, and Trp is a key pathway for the synthesis of the important neurotransmitter 5-HT. Abnormalities in the Kyn pathway may lead to decreased levels of 5-HT, which in turn may affect mood regulation and the development of depression [120]. In a mouse model of depression induced by long-term corticosterone injections, it was found that intestinal dysbiosis led to mitochondrial dysfunction and reduced neurogenesis in the hippocampal region of the brain, which in turn led to depressive behaviors [121]. Further studies have also shown that the development of depression is closely related to autophagy mechanisms in the GBA. For example, Zifan et al. showed that gut microbiota metabolites are important factors in CNS homeostasis, and they found that supplementation of propionic acid from differential gut microbiota metabolites to Alzheimer’s disease (AD) model mice contributed to the maintenance of in vivo and in vitro mitochondrial homeostasis through the enhancement of PINK1/Parkin-mediated mitophagy in the pathophysiology of AD [122]. Furthermore, for the CUMS mouse model, supplementation with the probiotic Lactobacillus plantarum CR12 not only rebuilt the composition of the gut microbiota but also increased the level of autophagy in the hippocampal region and significantly ameliorated anxiety and obsessive–compulsive-like behaviors in depressed mice [123]. Thus, it is evident that the interaction of gut microbiota metabolites with the autophagy pathway plays a key role in regulating brain function and improving depressive symptoms. By influencing the autophagy mechanism, the gut microbiota not only helps to maintain the functional homeostasis of the central nervous system but may also provide new targets for the treatment of depression.

### 4.4. Autophagy and HPA Axis Dysregulation in Depression

Recent studies have shown a close interaction between cellular autophagy and the HPA axis in the pathogenesis of depression. The dysfunction of the HPA axis is widely recognized as an important pathological features of depression, and it plays an important role in the onset and progression of depression. The HPA axis regulates the stress response in vivo through the hypothalamic–pituitary–adrenal pathway, and its overactivity is closely related to symptoms of depression [124]. Multiple stressors in the social environment can activate the HPA axis and further lead to its dysfunction, ultimately leading to significantly elevated cortisol levels, which not only induce neuroinflammation but may also cause long-term damage to the central nervous system [125]. There is a strong correlation between cortisol signaling and depression, and it has been shown that chronic glucocorticoid exposure leads to a decrease in the levels of BDNF mRNA and its proteins, as well as a decrease in the expression of its receptors, which can severely affect neuronal function [126]. In animal models of chronic stress exposure, HPA axis dysfunction is characterized by persistently elevated cortisol concentrations and abnormal changes in associated neuroendocrine factors [127]. Further studies have shown that the HPA axis is in a hyperactive state in a mouse model of chronic stress exposure and that salt-inducible kinase 1 (SIK1) plays an important role in this process. Specifically, SIK1 expression levels are up-regulated in the paraventricular nucleus of the mouse hypothalamus, which in turn drives the hyperactivation of the HPA axis by positively regulating the synthetic pathway of corticotropin-releasing hormone (CRH) [128]. Autophagy, as an important cellular self-protection mechanism, may be involved in the process of depression by regulating the function of the HPA axis or interacting with its dysregulation. It has been shown that autophagy levels are decreased in patients with depression, and this decrease is closely related to the activation state of the HPA axis. In depression, the abnormal activation of the HPA axis has been found to increase glucocorticoid secretion and, through it, to produce a link with cellular autophagy [129]. It was further found that aberrant activation of the HPA axis may exacerbate neuronal damage by reducing neuronal acidic vesicular organelles, decreasing lysosomal content and attenuating autophagy [130]. FK506-binding protein 51 (FKBP51) acts as a key regulator of the glucocorticoid receptor (GR), which forms complexes with molecules such as FKBP51 in the resting state, and when GR is bound, FKBP51 is released from the complex [131]. It has been shown that FKBP51 binds to Beclin-1, promotes Beclin-1 phosphorylation, and activates the autophagy pathway [132]. Therefore, the dysfunction of the HPA axis may lead to abnormalities in the autophagy process, further triggering neuronal damage and death. In addition, recent studies have revealed bidirectional regulation between autophagy and the HPA axis, and the restoration of autophagy function may regulate the overactivation of the HPA axis through a negative feedback mechanism [133]. For example, some experiments have shown that the level of CRH released by the HPA axis can be restored by attenuating autophagy disorders in a rat model of post-stroke depression, thereby preventing hippocampal synaptic loss and attenuating depressive-like behaviors [134]. In addition, autophagy plays a crucial role in maintaining neuroplasticity and neuroprotection by removing damaged proteins and injured mitochondria, which helps to attenuate the neurological damage associated with HPA axis overactivation [135]. In conclusion, the current study suggests that the overactivation of the HPA axis and defective autophagy may be important mechanisms in the pathogenesis of depression. Modulating the autophagy pathway may be a potential therapeutic strategy to alleviate symptoms associated with depression, while modulating the function of the HPA axis may also play a key role in autophagy recovery.

## 5. Autophagy Modulation as a New Strategy for the Treatment of Depression

### 5.1. mTOR Pathway Modulators

#### 5.1.1. Rapamycin and Its Analogs

Rapamycin and its analogs are first-generation mTOR inhibitors, and in depression, aberrant activation of the mTOR pathway may lead to the inhibition of autophagy, mitochondrial dysfunction, and the impairment of synaptic plasticity. Rapamycin forms a complex by binding to FK506-binding protein 12 (FKBP12), which in turn binds to the FRB structural domain of mTORC1, thereby inhibiting mTORC1 activity and deregulating its negative regulation of autophagy [136]. The classic autophagy inducer rapamycin has been found to have antidepressant-like effects, emphasizing the role of the mTOR pathway in this respect [137,138]. It has been shown that rapamycin reduces cellular focal death and ameliorates behavioral abnormalities in the hippocampus of mice in an animal model of H. pylori-induced depression [139]. Meanwhile, rapamycin inhibits NLRP1 inflammatory vesicle activation, ameliorates chronic social failure stress-induced depression-like behavior, and exhibits neuroprotective effects in mice with depression-like symptoms [53]. In addition, the rapamycin analog tesirolimus reduced depression-related resting time in a forced swimming test [137]. Meanwhile, in the hippocampus of LPS-induced depressed mice, rapamycin significantly enhanced the expression of LC3-II/I and Beclin-1 and significantly attenuated the expression of p62, suggesting the activation of autophagy [140]. Furthermore, rapamycin activated autophagy to prevent cognitive dysfunction and neuronal apoptosis in aged rats, and this effect was abolished by the neuroprotective activity of rapamycin following co-treatment with the autophagy inhibitor 3-methyladenine [141].

#### 5.1.2. Natural Products

Notably, natural products have superior anti-inflammatory properties in depression with greater efficacy and lower toxicity. Numerous natural products have been found to improve depression by modulating the mTOR pathway (Table 2). For example, apigenin, one of the most common flavonoids found in herbs and vegetables, was shown to alleviate depressive-like behaviors by activating the AMPK/mTOR pathway in CRS-stimulated mice treated with it, and improved levels of autophagy-associated molecules, such as LC3-II/I, as well as p62, were also found in the hippocampus [142]. In addition, PI3K/Akt/mTOR is also an important signaling pathway that regulates cellular autophagy. A modified formula based on the traditional Chinese medicine Xiaoyao San formula was found to inhibit the M1 phenotype of microglia in depressed mice by enhancing autophagy, and this effect was achieved by promoting the PI3K/Akt/mTOR signaling pathway [143]. Resveratrol is a naturally occurring polyphenol compound that affects a variety of cellular biological processes and has been shown to have benefits in modulating neurotransmitters and promoting neuroplasticity in psychiatric disorders such as depression [144]. Resveratrol supplementation was found to reduce the immobilization time of the FST and TST in CUMS mice to exert antidepressant effects [145]. In addition, resveratrol increased the expression of ATG5 and Beclin-1 but decreased the levels of p-Akt and p-mTOR in a model of mice with postpartum depression [146]. These findings suggest that the regulation of autophagy in depression by resveratrol may be related to the Akt/mTOR pathway. These findings suggest that natural products and traditional herbs may work together to coordinate the regulation of neural signaling and neuronal cell function as antidepressants with autophagy modulation by modulating mTOR pathway-mediated autophagy.

### 5.2. Mechanisms of Autophagy Regulation by Antidepressant Drugs

Antidepressant medication is a common and effective intervention in the management of depression. SSRIs, tricyclic antidepressants, serotonin and norepinephrine reuptake inhibitors, and monoamine oxidase inhibitors are widely used antidepressants in clinical practice. Among them, fluoxetine, the first SSRI, is widely used for its significant clinical efficacy and good safety [147,148,149]. It was found that in a model of depression induced by bilateral olfactory bulbectomy, fluoxetine treatment significantly alleviated abnormalities in the AMPK and mTOR signaling pathways and repaired the levels of LC3-II, Beclin-1, and p62 in the hippocampus of rats by promoting autophagy [67]. In addition, in a mouse model of chronic unpredictable stress (CMS)-induced autophagy, fluoxetine initiated mitophagy by enhancing the translocation of Parkin from the cytoplasm to the mitochondria, which, in turn, increased the expression of LC3 and autophagosome formation in the hippocampal region of mice [150]. Microglia, as resident immune cells in the CNS, have an important impact on the pathological process of depression in terms of regulating inflammation, synaptic plasticity, and hippocampal neurogenesis [151,152]. Especially after microglia activation, the increased inflammatory response can further damage neurons, so efficient autophagy mechanisms are especially critical for maintaining normal cell function [153]. Fluoxetine can restore autophagy function, inhibiting the levels of inflammatory cytokines in CUMS mice and CORT-activated microglia and normalizing the expression levels of Beclin-1, p62, and LC3 [154]. Meanwhile, in primary mouse microglia, fluoxetine also activated the autophagic pathway, as evidenced by elevated LC3 levels and increased lysosome formation [155]. Similarly, astrocytes, the most abundant glial cells in the CNS, have also been found to promote mitophagy in CMS mice and primary astrocytes, where fluoxetine was found to remove damaged mitochondria from the cells [150]. Sertraline, another SSRI, induces autophagy by decreasing intracellular ATP levels, thereby activating the AMPK/mTOR pathway, and this effect significantly inhibits the microtubule-associated protein tau [156]. A high-throughput screening study showed that sertraline promotes the accumulation of PINK1 protein, which triggers mitophagy and reduces mitochondrial membrane potential and ATP production, but its effect on the rate of oxygen consumption is not significant, which provides a new idea for improvement in symptoms associated with neurodegenerative diseases [157]. In addition, a variety of antidepressants such as paroxetine and amitriptyline rely on autophagy mechanisms to modulate metabolic imbalances and immune responses in patients or cellular models of depression, providing new insights into the clinical efficacy of depression [158]. 

**Table 2:** Effects of different types of antidepressant drugs on autophagy.

Drug		Experimental Design	Molecular Mechanism	Significance	Ref.
Natural products	Apigenin	Male BALB/c mice (*n* = 10), CSD for 21 days, Hip detected by WB	↑ LC3II/I, AMPK, ULK1 protein ↓p62, mTOR protein	↑ AMPK/ULK1/mTOR-mediated autophagy	[142]
	Modified Xiaoyao San formula	Male ICR mice (*n* = 10), LPS (1 mg/kg) for 2 weeks, PFC detected by WB and IF	↑ p62, ATG5 protein ↓ULK1, mTOR, PI3K, Akt protein ↑ LC3B-Iba-1 immunofluorescence colocalization	↑ PI3K/Akt/mTOR-mediated autophagy	[143]
	Resveratrol	Female C57BL/6 mice (*n* = 9), ovariectomized, underwent estradiol benzoate treatment, Hip detected by IF and WB	↑ LC3II/I, p62, ATG5, SIRT1 protein ↓p62 protein ↑ SIRT1 immunofluorescence	↑ SIRT1-mediated autophagy	[146]
Antidepressant	Fluoxetine	Male SD rats (*n* = 8), bilateral olfactory bulbectomy, Oral fluoxetine (10 mg/kg) for 30 days, Hip detected by WB	↑ LC3II, Beclin-1, AMPK, protein ↓p62, mTOR, ULK1 protein	↑ AMPK/mTOR-mediated autophagy	[67]
	Fluoxetine	Male C57BL/6 mice (*n* = 6), CMS for 5 weeks, oral fluoxetine (10 mg/kg) for 4 weeks, Hip detected by TEM and WB; primary astrocytes exposed to 1.2 mM CORT, cultured with fluoxetine (10 μM) for 24 h, cells detected by IF and WB	↑ Parkin protein ↓LC3II/I, p62, TOMM20 protein ↑ Autophagosome ↑ LC3–MitoTracker immunofluorescence colocalization	↑ Mitophagy	[150]
	Fluoxetine	Primary microglia cells cultured with fluoxetine (7.5 μM) and LPS (100 ng/mL) for 3 h, cells detected by IF and WB	↑ LC3-II protein ↑ LC3 immunofluorescence	↑ Autophagy	[155]
	Sertraline	Sertraline treatment of nematode strains expressing mCherry::LGG-1 and nematode strains expressing mitochondria-targeted GFP	↑ PINK1 protein ↑ Autophagosome and mitochondrial fragmentation	↑ PINK1-mediated mitophagy	[157]

Note: CSD, chronic stress depression; WB, Western blot; IF, immunofluorescence; Hip, hippocampus; PFC, prefrontal cortex; TEM, transmission electron microscope; ↑, increase; ↓, decrease.

## 6. Conclusions and Outlook

Depression, the most common mental disorder, affects the health of millions of people worldwide. Although the pathogenesis of depression is intricate, extensive research in recent years has found a strong association with the dysregulation of autophagy in the development of depression [159]. Cytosolic autophagy is a cellular degradation process that occurs in eukaryotic organisms and is essential for neuronal survival and function by removing damaged mitochondria and proteins and maintaining intracellular homeostasis [160]. Altered neuronal autophagy is thought to be involved in the pathogenesis of Alzheimer’s disease and Parkinson’s disease, which has broad prospects for research into and the clinical management of depression [161]. In addition, we reviewed the changes in autophagy observed in depression, suggesting that targeting autophagy may be a promising therapeutic avenue.

Indeed, although some research progress has been made in the mechanism of autophagy’s role in depression, many questions and challenges remain. Currently, the role of autophagy in depression is dual, as it may either promote the development of depression through overactivation or lead to the development of depression through inhibition. In addition, autophagy involves multiple signaling pathways and may intersect with depression-related pathological processes. However, current studies mostly focus on the single pathways of AMPK/mTOR and Akt/mTOR, and systematic analyses of multidimensional regulatory networks are lacking. Future studies should further delve into the specific mechanisms of autophagy’s role in depression, especially the interactions between autophagy and the dysregulation of neuroinflammation, neurogenesis, the gut microbiota, and the HPA axis. In addition, the development of novel antidepressant drugs based on autophagy modulation is promising. For example, activating autophagy by modulating the mTOR pathway or using natural products may provide new strategies for the treatment of depression.

## Figures and Tables

**Table 1:** Changes in autophagy observed in patients with depression and in animal and cellular models.

Species	The Model of Animals or Cells	Experimental Design	Behavioral Changes	Autophagy Changes	Significance	Ref.
Human		SnRNA-seq (*n* = 17) and transcriptome data validation from GEO		↓ LAMP2, LC3A, ATG4B, ATG9A, LC3B, ATG4D genes	↓ Autophagy initiation	[45]
		PB analyzed by RNA-seq (*n* = 17–19) and verified by qPCR (*n* = 32–33)		↓ LC3A mRNA		
		Analysis of postmortem PFC microarray data in GEO database (*n* = 29–56)		↑ ATG5, ATG6, ATG7, ATG12, LC3B mRNA ↓ p62 mRNA	↑ Autophagy initiation	[46]
		PB detected by RNA-seq (*n* = 4), qRT-PCR (*n* = 50), and ELISA (*n* = 44)		↑ p62 genes, mRNA and protein	↓ Autophagy degradation	[47]
		Patients with MDD and healthy volunteers (*n* = 10–14), detected by qPCR		↓ LC3A, NIX mRNA	↓ NiX-mediated mitophagy	[48]
Animal	LPS mice	Male C57BL/6 mice (*n* = 9), detected by IF and WB in the Hip	↓ Sucrose preference ↓ Number of crossings in OFT	↓ LC3 immunofluorescence ↓ LC3-II, Beclin-1 protein	↓ Autophagosome formation	[49]
	CORT mice	Male C57BL/6 mice (*n* = 12) exposed to CORT for 8 weeks, and hippocampal DG detected by IF, WB, confocal imaging, and TEM	↑ Immobility time in FST and TST ↓ Open arm time in EPM, central area time in OFT, exploration time in NORT	↑ LC3-II, ATG5 protein ↓ p62 protein ↑ Autophagosome, lysosome ↑ LC3-NeuN immunofluorescence colocalization ↑ Adeno-associated virus mCherry-GFP-LC3 fusion protein	↑ Autophagy	[8]
	CUMS mice	Male C57BL/6 mice (*n* = 6), CUMS for 6 weeks, brain tissue detected by qPCR and WB	↓ Sucrose preference ↑ Immobility time in FST	↓ Beclin-1, LC3 mRNA and protein ↑ p62, mTOR mRNA and protein	↓ Autophagy degradation	[51]
	CUMS mice	Female C57BL/6 mice (*n* = 8), CUMS for 8 weeks, Hip detected by IF, TEM, and WB	↓ Sucrose preference ↑ Immobility time in OFT, TST, FST ↑ Feeding latency in NSFT	↓ PINK1, Parkin, ATG5, LC3II/I protein ↓ Autophagosome ↓ PINK1 immunofluorescence	↓ PINK1/Parkin-mediated mitophagy	[52]
	CSDS mice	Male C57BL/6 mice (*n* = 9), CSDS for 10 days, Hip detected by TEM and WB	↓ Sucrose preference, total travel distance in OFT, social interaction rate in SIT ↑ Immobility time in TST, FST	↓ LC3-II/I, Beclin-1, ATG5, ATG7 protein ↑ Autophagosome ↑ p62, p-PI3K, p-AKT, p-mTOR protein	↓ PI3K/AKT/mTOR-mediated autophagy	[53]
	LH mice	Male ICR mice (*n* = 15), LH for 2 weeks, midbrain detected by WB	↑ Immobility time in FST, feeding latency in NSFT	↓ TSPO, PINK1, Beclin-1 protein ↑ Parkin protein	↓ TSPO-mediated mitophagy	[55]
Cell	BV2 cell exposed to LPS and ATP	Cultured with LPS (1 μg/mL) for 24 h and ATP (5 mM) for 30 min, cells detected by IF and WB		↓ LC3-II, PINK1, Parkin protein ↑ p62, TOM, TIM protein ↑ p62-TOM immunofluorescence colocalization	↓ Mitophagy degradation	[56]
	Primary astrocytes exposed to LPS	Cultured with LPS (1 μg/mL) for 24 h, cells detected by WB and confocal imaging		↓ LC3 protein ↑ p62 protein ↓ Adeno-associated virus GFP-mRFP-LC3 fusion protein	↓ Autophagy degradation	[57]
	HT22 cell exposed to CORT	Cultured with CORT (100 μM) for 24 h, cells detected by IF, qPCR, and TEM		↓ ATG5 immunofluorescence ↓ PINK1, Parkin, ATG5, LC3 mRNA ↓ Autophagosome	↓ Mitophagy	[52]

Note: SnRNA-seq, single-nucleus RNA sequencing; qPCR, quantitative polymerase chain reaction; GEO, gene expression omnibus; PB, peripheral blood; RNA-seq, RNA sequencing; Hip, hippocampus; PFC, prefrontal cortex; qRT-PCR, quantitative reverse transcription PCR; MDD, major depressive disorder; WB, Western blot; IF, immunofluorescence; DG, dentate gyrus; CORT, cortistatin; TEM, transmission electron microscope; CUMS, chronic unpredicted mild stress; CSDS, chronic social defeat stress; LPS, lipopolysaccharide; LH, learned helplessness; ATP, adenosine triphosphate; OFT, open-field test; FST, forced swimming test; TST, tail suspension test; EPM, elevated plus maze; NORT, novel object recognition test; NSFT, novelty suppressed feeding test; SIT, social interaction test; ↑, increase; ↓, decrease.

## Data Availability

Not applicable.

## Abbreviations

HPA, hypothalamic–pituitary–adrenal; SSRIs, selective serotonin reuptake inhibitors; CMA, chaperone-mediated autophagy; Hsc70, Heat Shock Cognate 70; ATG, autophagy-associated gene; mTORC 1, mammalian target of rapamycin complex 1; ULK 1, unc 51-like autophagy-activated kinase 1; PI3P, phosphatidylinositol 3-phosphate; VPS34, vesicular protein sorting 34; PE, phosphatidylethanolamine; LAMP2A, lysosome-associated membrane protein 2A; HFD, high-fat diet; ESCRT, endosomal sorting complex required for transport; MDVs, mitochondria-derived vesicles; PINK1, PTEN-inducible kinase 1; OMM, outer mitochondrial membrane; SQSTM1, sequestosome 1 also known as p62; OPTN, Optineurin; NDP52, Nuclear Dot Protein 52; LIR, LC3-interacting region; NIX, Nip3-like protein X; BNIP3, Bcl2-interacting protein 3; FUNDC1, FUN14 structural domain-containing 1; GABARAP, γ-aminobutyric acid receptor-associated protein; HIF-1α, hypoxia-inducible factor 1α; NTRK1, neurotrophic tyrosine kinase receptor 1;MAPK, mitogen-activated protein kinase; CUMS, chronic unpredictable stress; ROS, reactive oxygen species; MKP-1, mitogen-activated protein kinase phosphatase-1; LPS, lipopolysaccharide; DSS, dextran sodium sulfate; BDNF, brain-derived neurotrophic factor; NBR1, Neighbor of BRCA1 gene 1; CSDS, chronic social defeat stress; CORT, corticosterone; LH, learned helplessness; TSPO, translocator protein; VDAC1, voltage-dependent anion channel; NSCs, neural stem cells; NRBF2, Nuclear Receptor-Binding Factor 2; GBA, gut–brain axis; FKBP51, FK506-binding protein 51; GR, glucocorticoid receptor; SIK1, salt-inducible kinase 1; CRH, corticotropin-releasing hormone.
